# Recent HIV-1 Infection: Identification of Individuals with High Viral Load Setpoint in a Voluntary Counselling and Testing Centre in Rural Mozambique

**DOI:** 10.1371/journal.pone.0031859

**Published:** 2012-02-21

**Authors:** Celia Serna-Bolea, Nilsa de Deus, Sozinho Acácio, Jose Muñoz, Delino Nhalungo, Emilio Letang, Pedro Alonso, Denise Naniche

**Affiliations:** 1 Barcelona Centre for International Health Research (CRESIB, Hospital Clínic-Universtitat de Barcelona), Barcelona, Spain; 2 Manhiça Health Research Centre (CISM), Manhiça, Mozambique; 3 National Institute of Health, Maputo, Mozambique; Istituto Superiore di Sanità, Italy

## Abstract

**Background:**

Identification of recent HIV-infections is important for describing the HIV epidemic and compiling HIV-RNA-setpoint data for future HIV intervention trials. We conducted a study to characterize recent infections, and HIV-RNA-setpoint within the adult population presenting at a voluntary counselling and testing centre (VCT) in southern Mozambique.

**Methods:**

All adults attending the Manhiça District-Hospital VCT between April and October 2009 were recruited if they had at least one positive rapid HIV-serology test. Patients were screened for recent HIV-1 infection by BED-CEIA HIV-incidence test. Clinical examination, assessment of HIV-RNA and CD4 cell counts were performed at enrollment, 4 and 10 months.

**Results:**

Of the 492 participants included in this study, the prevalence of recent infections as defined by BED-CEIA test, CD4 counts >200 cells/µl and HIV-RNA >400 copies/mL, was 11.58% (57/492; 95% CI 8.89–14.74). Due to heterogeneity in HIV-RNA levels in recently infected patients, individuals were categorized as having “high” HIV-RNA load if their HIV-RNA level was above the median (4.98 log_10_ copies/mL) at diagnosis. The “high” HIV-RNA group sustained a significantly higher HIV-viral load at all visits with a median HIV-RNA setpoint of 5.22 log_10_ copies/mL (IQR 5.18–5.47) as compared to the median of 4.15 log_10_ copies/ml (IQR 3.37–4.43) for the other patients (p = 0.0001).

**Conclusion:**

The low proportion of recent HIV-infections among HIV-seropositive VCT clients suggests that most of this population attends the VCT at later stages of HIV/AIDS. Characterization of HIV-RNA-setpoint may serve to identify recently infected individuals maintaining HIV viral load>5 log10 copies/mL as candidates for antiretroviral treatment as prevention interventions.

## Introduction

HIV prevention strategies in Sub-Saharan Africa have been mainly focused on voluntary testing and counselling centres (VCT) and condom promotion. However, these efforts have shown limited success in controlling the HIV epidemic and the number of infected people continues to increase, having reached 33.3 million [31.4–35.3 million] people living with HIV in 2009 [1]. Current rapid serology tests employed in most resource-poor countries do not differentiate between acute, recent and longstanding HIV infection which have different risks of transmission.

During the early months of HIV infection, levels of HIV-RNA in plasma and genital secretions are up to 2 log_10_ higher than during the chronic phase and only comparable to end stage AIDS [2], [3]. Indeed, each log_10_ increase in HIV viral load has been shown to be associated with a 2.45 increased risk of heterosexual HIV transmission [4], [5]. However, initial peak of HIV viremia during acute HIV infection usually decreases within 6–8 weeks and then gradually over six months to a more stable level, often referred to as the viral load setpoint [6]. The level at which the HIV-RNA setpoint is established is predictive of both disease progression and the probability of HIV transmission during chronic HIV infection [3], [7]. Studies have suggested that 50% of HIV transmissions take place in the first 6 months after infection when HIV-RNA levels can be up to 2 log_10_ higher than during chronic HIV infection [5], [8]. In this view, some authors suggest that antiretroviral treatment and/or behaviour modification interventions focused in the initial phases of infection when HIV-RNA levels are elevated could help in reducing HIV transmission [9], [10]. Recently, it has been proposed that individuals maintaining high HIV-RNA levels during the initial phases of infection could be targeted for interventions to reduce HIV transmission [10]. In line with this, early initiation of antiretroviral treatment (ART) has recently been shown to reduce sexual transmission of HIV among HIV serodiscordant couples [11]. Moreover, it has been predicted that a vaccine aimed at decreasing viral load setpoint could be an effective strategy to prevent new HIV infections and progression to AIDS [12], [13].

In the last ten years many serological assays have been developed to distinguish between recent infections (<6 months since seroconversion) and established HIV infections [14]. These assays have been used to assess HIV-RNA setpoint as well as estimate HIV incidence in a cross-sectional manner [14], [15], [16], [17].

The most widely used assay to determine recent infections is the BED capture enzyme-immunoassay (BED-CEIA), frequently applied in developing countries [18], [19], [20], [21], [22], [23]. However, the assay may lead to misclassification of advanced AIDS as recent infections [24]. To avoid these misclassifications they are usually validated for the population screened, and the results preferably adjusted for False Positive Rates (FPR) [25].

Characterization of recent HIV infections in African populations can aid in estimating the proportion of the population accessing early diagnosis of HIV, as well as determining HIV viral load setpoint levels. Indeed description of the epidemiology of recent HIV infections may inform programs seeking to improve VCT uptake. Additionally, identifying those individuals more likely to transmit HIV could be useful for positive HIV prevention efforts. Finally, assessing the HIV-RNA setpoint contributes to understanding the dynamics of HIV transmission in a population that is predominantly infected with HIV subtype C [26], which has been reported to have higher levels of HIV-RNA during the acute phase of HIV infection [27]. Little is known about the evolution of infection for this subtype.

The objective of the study was to characterize recent infections and HIV viral load setpoint within the HIV-seropositive adult population presenting at a VCT in southern Mozambique.

## Materials and Methods

### Study population and visits

All adults attending the VCT of the Manhiça District Hospital (MDH) in Manhiça, southern Mozambique, between April and October 2009 were recruited if the first Determine (Abbott Park Illinois, USA) serological rapid test was positive, and the confirmatory rapid test Unigold (Trinity Biotech Co., Wicklow, Ireland) was positive or negative or indeterminate, and if they were permanent residents of the demographic surveillance study area. During the study, approximately 450 individuals attended the VCT monthly, of which an estimated 39% were residents of the demographic surveillance study area.

The Manhiça Health Research Centre (CISM) has been conducting continuous demographic surveillance in the district since 1996 which covered a population of 82,000 persons at the time of this study.

Participants were screened for recent HIV-1 infection by BED-capture enzyme immunoassay incidence assay (BED-CEIA). CD4 T cell counts were performed for all participants, and plasma was frozen for subsequent determination of HIV-RNA viral load if BED-CEIA identified them as recently infected. Patients identified as recently infected were invited for follow-up visits at 4 and 10 months for CD4 and CD8 T cell counts and HIV-RNA assessment. Clinical examination and epidemiological data were recorded at each visit.

During the study period, six patients became eligible for ART and initiated treatment (three at 4 months and another three at 10 months.). Those patients were excluded from the study when they initiated antiretroviral therapy. In accordance with the Mozambican National Antiretroviral Treatment Guidelines, ART initiation criteria were defined as CD4+ cell count ≤200 cells/mL irrespective of the clinical stage, WHO stage III with CD4cell count ≤350 cells/ml, or WHO stage IV irrespective of CD4 cell count.

Written informed consent was obtained from patients prior to participation. The study protocol was reviewed and approved by the Mozambican National Bioethics Committee (ref. 232/CNBS/07) and the Hospital Clinic of Barcelona Ethics Review Committee (ref. CEIC/2007/3943).

### Laboratory procedures

#### BED enzyme immunoassay

Recent infections were determined from cryopreserved plasma samples with the commercial BED-capture enzyme immunoassay (BED-CEIA) strictly following the manufacturer's instructions (Calypte Biomedical Corporation, Portland, OR 97224).

The BED-CEIA is a quantitative antibody assay that determines the proportion of HIV-1-specific IgG antibodies in samples with respect to total IgG antibodies; BED-CEIA detects recent seroconversion as samples presenting low levels of HIV-1-specific IgG considered to reflect infection within a window of approximately 6 months [15]. Seropositive samples from individuals testing below the threshold of a normalized optical density (ODn) of less than 0.8 are classified as recently infected, likely to have been infected within the past 6–8 months.

#### HIV-RNA determinations

HIV-RNA levels were determined from cryopreserved plasma samples with the commercial Roche Amplicor Monitor, version 1.5 (Roche Diagnostics, Basel, Switzerland) technique for amplification and quantification of HIV-1 RNA. Lower limit of detection was 400 copies/mL. For the purpose of analyses, plasma HIV-1 RNA concentrations below the limit of detection were assigned the value of 200 copies/mL.

#### CD4 and CD8 counting

CD4 and CD8 counting was performed after staining with labelled antibodies: CD4, CD3, CD8, and CD45 in TruCount tubes (Becton Dickinson Biosciences, San Jose, California, USA). Samples were assessed by flow cytometry on a FACS_Calibur (Becton Dickinson).

### Statistical analysis and definitions

Proportions for categorical variables were compared using the Pearson chi-squared test. The Wilcoxon rank-sum test was used to compare medians of continuous variables with non-normal distribution and the Wilcoxon signed-rank test to compare paired groups. Statistical analyses were performed using STATA version 11 (STATA Corp., College Station, Texas, USA).

Patients were considered HIV-infected when both the first (Determine, Abbott Park Illinois, USA) and the confirmatory (Unigold, Trinity Biotech Co., Wicklow, Ireland) rapid HIV serology tests were positive, and indeterminate when only the first rapid test was reactive.

Recently infected patients were defined as those individuals with HIV-positive or indeterminate serology (as defined above) and a BED-CEIA assay ODn reading of <0.8, CD4 counts >200 cells/µl and HIV-RNA levels >400 copies/mL. All recent-BED-CEIA testing patients with CD4 counts below 200 cells/µl or Viral Load <400 copies/mL were considered long-standing infected patients and those with low CD4 counts (<200 cells/µl) referred for ART initiation. CD4 count <200 cells/µl and viral load (VL) <400 copies/mL have been associated with misclassification of recent infections by BED-CEIA [28].

Those patients categorized as having a “high” HIV-RNA load were defined as those individuals with HIV-RNA level at enrollment above the median HIV-RNA level (4.98 log_10_ copies/mL).

Assuming that the viral load setpoint is established during the first year of infection, we defined that viral load setpoint had been reached in a population when the median HIV-RNA measurements did not show significant differences between visits. The viral load setpoint of each individual was then calculated as the mean of at least two consecutive measurements. The population viral load setpoint was expressed as median and interquartile range (IQR).

## Results

### Characteristics of the study population

Among 492 individuals enrolled in the study who presented at the Manhiça hospital VCT with a positive HIV Determine rapid test, 97.7% (481/492) had a confirmed HIV positive serology and 2.23% (11/492) had an indeterminate HIV serology, as defined in methods (Table 1).

**Table 1:** HIV serological characteristics of the study population (N = 492).

	n	% (95%CI)
**HIV RT positive**	481	97.7 (95% CI 96.03–98.87)
**HIV RT indeterminate**	11	2.23 (95% CI 1.12–3.96)
**BED-CEIA-recent**	80	16.26% (95% CI 13.10–19.82)
**BED-CEIA-recent, CD4>200 cell/µl**	61	12.39% (95% CI 9.61–15.63)
**BED-CEIA-recent, CD4>200 cell/µl, VL>400 copies/mL**	57	11.58% (95% CI 8.89–14.74)

RT: rapid testing. BED-CEIA: BED capture enzyme immunoassay. VL: Viral Load.

HIV positive and indeterminate serology by RT and BED-CEIA recent infection as defined in methods.

The median age of the study population was 34 years (IQR 28–42) ranging from 18 to 86 and patients were predominantly female (69.30%; 341/492). Women were significantly younger than men [median: 33 years (IQR 27–40) versus 35 years (IQR 29–46), respectively, p = 0.0012].

### Epidemiology of recent HIV infection

Of the 492 individuals enrolled, 80 [16.26%; 95% CI 13.10–19.82] were classified as having a recent infection by the BED-CEIA assay (Table 1). Of these 80 patients, 19 (23.75%) had CD4 counts below 200 cells/µl and were considered to be advanced HIV infections, according to the definitions applied (see methods). In addition, four individuals (6.55%) with VL<400 copies/mL (6.55%) were also considered long-standing infections and excluded from the recently infected population in order to reduce the rate of misclassification. Thus, using CD4<200 cells/µl as a surrogate of false positivity, the FPR for recent infections was 3.86%, and adding VL<400 copies/mL to the definition, the FPR for recent infections was 4.67%. As a consequence, the prevalence of recent HIV infection was 12.39% (61/492), (95% CI 9.61–15.63) or 11.58% (57/492), (95% CI 8.89–14.74), as defined by the BED-CEIA assay and CD4 counts greater than 200 cells/µl or BED-CEIA and CD4 counts greater than 200 cells/µl and VL greater than 400 copies/mL respectively. The remaining 435 (88.41%) patients were classified as having a long-standing HIV-1 infection. Due to the unknown validated FPR for BED-CEIA in our population, we did a sensitivity analyses to assess the impact of different FPR on the population prevalence of recent infections. We randomly assigned FPR of 1%, 3% and 5% to the BED-CEIA assay in our study population based on values reported in other countries [22], [23], [24]. These calculations yielded prevalences of recently infected patients of 15.26%, 13.26% and 11.26% respectively. These values fell within close range of the confidence interval of the prevalence of recent infections determined using CD4<200 cells/µl and VL<400 copies/mL as surrogates of FPR [11.58%, (95% CI 8.89–14.74)]. The remaining analysis was performed using the CD4-VL corrected definition of recent HIV infection.

Median HIV-RNA measurements and CD4 counts of BED-CEIA diagnosed recently infected individuals using the CD4-VL corrected definition are shown in table 2. Using the CD4-VL corrected definition of recent HIV infections, among those patients with confirmed HIV positive serology, 10.22% (95% CI 7.66–13.29) had a recent infection whereas among those with an indeterminate HIV serology, 88.88% (95% CI 51.75–99.71) had a recent infection.

**Table 2:** Baseline virological and immunological parameters among individuals diagnosed with recent HIV infections by the BED-CEIA assay.

	n	Median (IQR)
**BED, CD4>200 cells/µL**		
HIV-RNA log_10_ copies/mL	57	4.98 (4.29–5.44)
CD4 cells/µl	52*	516.5 (305–722)
**BED, CD4 <200 cells/µL or VL<400 copies/mL**		
HIV-RNA log_10_ copies/mL	23	5.23 (4.65–5.64)
CD4 cells/µl	23	135 (46–186)

Results are presented according to CD4 counts.

*CD4 counts available for 52/57 patients.

Women had a significantly greater proportion of recent HIV infections as compared to men (13.78% [95%CI 10.30–17.90] vs. 6.62% [95%CI 3.22–11.84] respectively, p = 0.022).

The median age of the recently HIV infected population was 34.0 years (IQR 24.9–46.9), similar to the age of the long-standing infected population [33.5 years (IQR 27.6–41.5), p = 0.9]. Within the recently infected population, the median age for women was 33.3 years (IQR 23.9–41.4), significantly younger than the median age for men [47.4 years (IQR 30.6–54.5), p = 0.030].

No patient self-reported specific symptoms characterized the HIV-1 recent infections in the population nor distinguished them from long-standing HIV infections (Table 3).

**Table 3:** Self-reported clinical symptoms according to status of HIV infection.

Reported symptom	Recent HIV infection*(n = 57) n (%)	Long-standing HIV infection (n = 435) n (%)	P-value
Loss of weight	32 (56.14)	277 (63.82)	0.30
Cough	20 (35.09)	176 (40.46)	0.47
Diarrhea	3 (5.26)	43 (9.89)	0.33
Common Cold	15 (26.32)	141 (32.41)	0.45

*Recent HIV infection defined by BED-CEIA, CD4 counts >200 cell/µl and VL>400 copies/mL.

### Immuno-virological parameters in Recent HIV infected patients

Patients considered to be recently infected by HIV were followed up for assessment of their CD4 counts and HIV-RNA load. Median CD4 cell count measures at 4 months (497 cells/µl (IQR 347–597) and at 10 months (582 cells/µl (IQR 533–758) were not significantly different compared with values at HIV diagnosis [(516.5 cells/µl (IQR 305–722), p = 0.700 and p = 0.139 respectively].

Follow-up measurements of HIV-1-RNA in recently infected individuals showed a median viral load of 4.50 log_10_ copies/ml (IQR 3.88–5.16) at 4 months post-diagnosis which was slightly lower than that observed at diagnosis [4.98 log_10_ copies/ml (IQR 4.29–5.44), p = 0.044]. At 10 months post-diagnosis, median viral load was 4.88 log_10_ copies/ml (IQR 4.55–5.16), which was not significantly different from values at baseline or at the 4 month visit (p = 0.77, p = 0.34 respectively).

**Figure 1:**
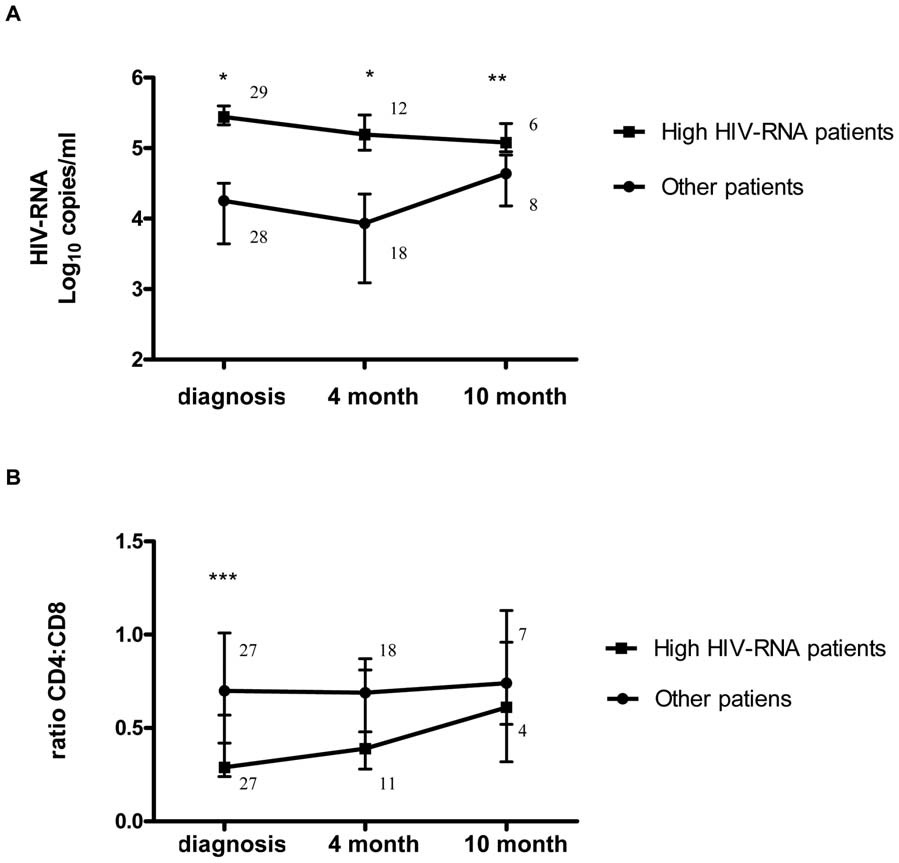
Evolution of median HIV-RNA load and CD4∶CD8 ratios in recently infected patients. HIV-RNA load (A) and CD4∶CD8 ratios (B) in patients with high HIV-RNA as compared to the other patients. Median and interquartile range are shown. HIV-RNA viral load groups are defined as described in methods. P-values are from Wilcoxon rank sum test between groups as follows: *p = 0.0001, **p = 0.038, *** p = 0.002. P-values from Wilcoxon Signed Rank between visits did not show significant differences. Numbers denote the n for each group at each time point.

Because of great heterogeneity in patterns of HIV-RNA evolution over time, recently infected patients were categorized as having “high” HIV-RNA load if the HIV viral load at diagnosis was above the median (described in methods). The group of patients with high HIV-RNA sustained a significantly higher median HIV viral load at all visits compared with the other patients (Figure 1A). The interquartile ranges showed that those individuals with high HIV-RNA levels had a lower intra-group heterogeneity in HIV-RNA levels than did the other patients. Within the group of patients with high HIV-RNA, a stable viral load was maintained from diagnosis through to the 10 month follow-up with no statistically significant differences in HIV-RNA levels between visits (Figure 1A). Thus, individual HIV viral load setpoint values were determined by pooling HIV-RNA data as described in methods. The median population HIV-RNA setpoint for the high HIV-RNA patients was 5.22 log_10_ copies/ml (IQR 5.18–5.47) as compared to 4.15 log_10_ copies/ml (IQR 3.37–4.43) for the other patients (p = 0.0001).

Median CD4 counts did not differ significantly between the high HIV-RNA group and the other patients for all follow-up visits. Median CD8 counts were increased in those with high HIV-RNA levels for all follow-up visits, but a significant difference between groups was only observed at diagnosis (high HIV-RNA group 1152 (IQR 900–1893) vs. the other patients 894 cells/µl (IQR 605–1379), p = 0.031). In addition, the CD4∶CD8 ratio at diagnosis was lower in the high HIV-RNA group as compared to the other patients (p = 0.0027) (Figure 1B).

## Discussion

Our findings show that 11.58% of clients of the Manhiça Hospital VCT in southern Mozambique may be considered to have a recent HIV infection. There was a higher prevalence in women than in men. Assessment of HIV viral load setpoint in recently infected individuals revealed great heterogeneity of HIV-RNA viral load from which two patterns emerged. Individuals with HIV viral load above the median at enrollment (>4.98 log_10_ copies/mL) sustained a high HIV-RNA level and a setpoint of 5.22 log_10_ copies/mL. This contrasted with individuals with an initial HIV viral load below the median and a viral load setpoint of 4.15 log_10_ copies/mL.

The prevalence of recently infected HIV patients (11.58%) among HIV-seropositive VCT clients in Manhiça was similar to that of other African countries [19] but lower than that observed in European countries such as Switzerland where a prevalence of 37% of recent HIV infections was reported in VCT clients as determined by the BED-CEIA [29]. A low prevalence of recent infections suggests that a large proportion of the Manhiça population attends the VCT at later stages of HIV/AIDS. Thus, recently infected individuals, who are more likely to transmit, may have lower VCT uptake. This does not include pregnant women who undergo HIV testing in the Manhiça antenatal clinic (ANC). Clients of the ANC are younger with a median age of 24 years and may have a higher prevalence of recent HIV infections than those attending the general VCT [30], [31], [32]. Late diagnosis of HIV has been observed in other African countries, and it is likely due to a poor access to health care in these settings [33]. Late presentation is associated with a higher mortality after ART initiation [34] and this has led to piloting new HIV testing approaches to increase uptake, including mobile units, provider-based and home-based counselling and testing [35].

The gender and age distribution of recent HIV infection observed in our study is likely to be a reflection of the HIV epidemic and trends in VCT uptake in the area. We observed that the prevalence of recent HIV infections was significantly higher in women as compared to men, corroborating findings of increased HIV prevalence in women in sub-Saharan Africa [1], [36]. In our study, recently infected men were significantly older, almost 10 years when comparing with recently infected women. This may reflect migration trends for young healthy men from Manhiça working in South Africa [37]. This would most probably lead to underrepresentation of young men in the recently infected population, and overrepresentation in more advanced stages of disease when HIV progression may impede seeking employment in South Africa. These age and gender distributions suggest that other approaches to HIV testing are needed to increase earlier diagnosis of HIV in all age groups with particular attention to VCT uptake in men.

In terms of clinical signs and symptoms that could potentially differentiate recent from long-standing HIV infections, we did not observe any non-specific self-reported signs associated with either. It has been shown that nonspecific flu-like syndromes may help to identify patients during the acute phase of the infection which lasts up to 6 weeks [38], [39], [40], [41]. However, although patients may have high HIV viral loads over a longer period of time, nonspecific patient-reported symptoms do not appear to distinguish recent from longstanding HIV infected patients in our population. This study did not include clinical exploration for opportunistic infections.

HIV viral load setpoint has been loosely defined as a stable viral load level established within the first year after seroconversion [6]. However, in the literature the establishment time of this HIV viral load setpoint has been defined at various time points ranging from 4 months to 24 months post-infection [7], [42], [43], [44]. We estimated the HIV viral load setpoint over the 1^st^ year of HIV infection. In our study population, HIV-RNA levels of patients classified as recently infected remained elevated throughout the follow up. In addition, those with HIV-RNA levels greater than the median (4.98 log_10_ copies/mL) at diagnosis maintained HIV-RNA levels above 4.98 log_10_ copies/mL during the first year after infection with an estimated viral load setpoint of 5.22 log_10_ copies/mL. These individuals may be more rapid progressors and could have an increased risk of transmitting HIV. There is indeed agreement that higher HIV-RNA setpoints are associated with faster progression to AIDS and increased risk of HIV transmission [7], [45], [46], [47]. Individuals with HIV viral load above 4.91 log_10_ copies/mL have been suggested to progress to AIDS as quickly as 3 years after infection [48], and those patients with HIV-RNA levels above 4.7 log_10_ (50000) copies/mL may have the highest HIV transmission rates [4].

It has recently been suggested that individuals maintaining HIV-RNA viral loads greater than 4.7 log_10_ (50000) copies/mL could be a target for antiretroviral treatment for prevention to reduce HIV transmission [10]. The percentage of individuals with HIV viral loads above 4.7 log_10_ copies/mL in Botswana was suggested to be approximately 25%–30% both in the general population and in antenatal clinics tested by rapid serology tests [10]. According to this approach, up to 50% of recently infected individuals in the present study could be candidates for antiretroviral treatment for prevention and suggests that screening for recent HIV infections may identify a greater proportion of individuals with elevated viral load.

Our results showed that patients with elevated viral load had higher CD8 T-cell counts and lower CD4∶CD8 T-cell ratios at diagnosis. Other studies have suggested that within HIV-1 subtype C infected patients, those individuals who maintain a higher viral load setpoint display lower levels of CD4 cells [27]. We did not observe these low levels of CD4 counts in the group maintaining higher HIV-RNA viral load, however these patients showed decreased CD4∶CD8 ratios over the year of follow-up.

This study had several limitations. Although the BED-CEIA assay is widely used tool, it has not been validated for subtype C. It has been suggested that the BED-CEIA period of recency may be wider for subtype C than for subtype B. The recency window for subtype B is 162 days and could be up to 203 days for subtype C [49]. Nevertheless, several studies have used BED-CEIA for measuring recent infections and estimating incidence in countries where HIV subtype C is the major subtype [18], [19], [21], [22], [25], [50]. We considered a broader recency window of up to 8 months. However it is important to emphasize that the present study did not have the aim of measuring incidence but only a prevalence of recent infections in the area.

Another limitation is that BED-CEIA is known to overestimate the proportion of recently infected individuals which may lead to misclassification of advanced AIDS as recent infections [51]. To avoid this misclassifications a false positive rate (FPR) can be calculated for each population studied. However, the calculation requires comparison with longitudinal incidence studies, which were not available for the Manhiça population. We thus used CD4 counts and HIV-RNA levels as surrogates of FPR. Hence, our results were based on the assumption that BED<0.8n O.D., CD4>200 cells/µl and VL>400 copies/mL identifies recent infection. We, as other authors, considered those individuals reading as recent HIV infections by BED-CEIA but with CD4<200 cells/µl or VL<400 copies/mL as false recent infections [18], [28], [50], [52]. This is based on the assumption that the production of HIV-specific IgG decreases when viral replication is suppressed or in the presence of severe immunosuppression. This in turn can lead to a low proportion of specific HIV-IgG relative to total IgG giving a false recent infection by the BED-CEIA [28]. Nevertheless, we cannot exclude that a small number of advanced AIDS patients with CD4>200 cells/µl or VL>400 copies/mL were misclassified as recent infections which could lead to an overestimation of both the prevalence of recent infected patients and median HIV-RNA levels. On the other hand, the potential exclusion of recently infected patients with CD4<200 cells/µl or VL<400 copies/mL could lead to underestimating the prevalence of recent infected patients. However this is less likely. Finally, the study suffered a large loss to follow-up during the 10 months of the study leading to a low sample size at 10 months of followup. However, the loss was equal in both high HIV-RNA patients and those with lower HIV-RNA levels.

In addition to the use of BED-CEIA and FPR, other approaches such as antibody avidity tests or other detuned ELISA have been used to estimate recent infections alone or in combination [24], [25], [52]. The use of a second avidity test to identify recent infections diminishes the likelihood of misclassifications [24], however it may require specific technology which can limit its use in a developing country. The approach of using the BED-CEIA assay and CD4<200 cells/µl and VL<400 copies/mL as surrogate markers for FPR may facilitate the identification of recent infections in situ using the HIV monitoring tools already available or being scaled up in many low income countries.

In summary, the present study provides data on recent HIV infections and viral load setpoint from an area with little previous information. Our results suggest that in a rural area of southern Mozambique, a low proportion of individuals seek HIV testing at early phases of HIV infection and points to a need for new approaches to HIV testing. Our results also identify a group of patients in early phases of HIV infection with an elevated HIV-RNA setpoint greater than 5.0 log_10_ copies/mL which could be target for ART for prevention strategies.
